# Combined Transcriptomic Analysis Revealed AKR1B10 Played an Important Role in Psoriasis through the Dysregulated Lipid Pathway and Overproliferation of Keratinocyte

**DOI:** 10.1155/2017/8717369

**Published:** 2017-10-24

**Authors:** Yunlu Gao, Xuemei Yi, Yangfeng Ding

**Affiliations:** Shanghai Skin Disease Hospital, Tongji University School of Medicine, Shanghai 200050, China

## Abstract

RNA-seq has enabled in-depth analysis of the pathogenesis of psoriasis on the transcriptomic level, and many biomarkers have been discovered to be related to the immune response, lipid metabolism, and keratinocyte proliferation. However, few studies have combined analysis from various datasets. In this study, we integrated different psoriasis RNA-seq datasets to reveal the pathogenesis of psoriasis through the analysis of differentially expressed genes (DEGs), pathway analysis, and functional annotation. The revealed biomarkers were further validated through proliferation phenotypes. The results showed that DEGs were functionally related to lipid metabolism and keratinocyte differentiation dysregulation. The results also showed new biomarkers, such as AKR1B10 and PLA2G gene families, as well as pathways that include the PPAR signaling pathway, cytokine-cytokine receptor interaction, alpha-linoleic acid metabolism, and glycosphingolipid biosynthesis. Using siRNA knockdown assays, we further validated the role that the AKR1B10 gene plays in proliferation. Our study demonstrated not only the dysfunction of the AKR1B10 gene in lipid metabolizing but also its important role in the overproliferation and migration of keratinocyte, which provided evidence for further therapeutic uses for psoriasis.

## 1. Introduction

Psoriasis is a common inflammatory skin disorder that appears as demarcated chronic erythematous plaques covered with silvery white scales. Ethnic factors, genetic loci, and infections may contribute to the etiology of psoriasis [1, 2]. However, increasing evidence has shown that the disturbance of lipid metabolism coupled with a disrupted immune system and overproliferation of keratinocyte play an important role in the pathogenesis of psoriasis [3–5].

It is believed that abnormal fat metabolism is an important factor in the etiopathogenesis of psoriasis [5]. Research has shown that total lipids, phospholipids, triacylglycerols, and cholesterol are increased in the blood and epidermis of psoriasis patients [6]. Changes in the composition of phospholipids in psoriatic foci are reportedly involved in inflammation, congestion, and parakeratosis due to lipid deposition in the reticular-endothelial system [5]. Furthermore, continuous loss of psoriatic scales exacerbates the disease, and serum lipids are also affected by changes in skin lipids [4]. Decreased short-chain fatty acids levels in psoriatic and unaffected skin were observed using gas liquid chromatography [7]. The severity of psoriasis corresponds with the increased levels of free cholesterol and phospholipids in the epidermis [8].

Immune microenvironment disorders also contribute to the pathogenesis of psoriasis [9]. Currently, it is recognized that IL-23/17 dysregulation is involved in the immunopathology of psoriasis [10]. A sustained elevation of IL-23 can be found in the dendritic cells residing in the dermis, leading to Th-17 releasing IL-17 and IL-22, which further contribute to the overproliferation of keratinocytes [11–13]. Furthermore, continuous cytokines, chemokines, and antimicrobial peptides are released to chemoattract diverse immune cells, which amplify immune reaction, including the synthesis of CCL20, CXCL1, CXCL2, and CXCL8/IL-8 [14–16].

More recently, interactions between lipid metabolism and immune disorders and overproliferation of keratinocytes were found to be involved in the pathogenesis of psoriasis [17]. Di Fusco et al. have reported that overexpression of Smad7 could increase keratinocyte cell proliferation. Hermann et al. have reported that miR-146a and miR-146b synergically suppressed keratinocyte proliferation and inflammatory responses in psoriasis cases, which demonstrated the capability of modulating inflammatory responses and keratinocyte proliferation in psoriatic skin [18]. More recently, Di Fusco et al. have shown that keratinocyte overproliferation can be inhibited by acitretin through JAK/STAT signaling pathways to ameliorate psoriasis [19]. However, studies of keratinocyte overproliferation were based on a single or few known or plausible biomarkers, and there are no comprehensive analyses of the pathogenesis of psoriasis from the whole transcriptomic level [20].

In this study, we performed a combined analysis of psoriasis RNA-seq datasets to examine the roles lipids play in the immunopathogenesis of psoriasis. Differentially expressed genes, their functional analyses, and the expression of genes related to lipid metabolism were analyzed in this study, which demonstrated new biomarkers and pathways, such as the AKR1B10 and PLA2G gene families. To further determine whether the above biomarker (AKR1B10) also played an important role in the overproliferation of the psoriatic epidermis, we further explore the proliferation and migration roles of AKR1B10 using RNA interference in keratinocyte cell lines.

Our study provided a comprehensive analysis of the expression pattern of psoriasis, revealed the function and pathway change of psoriasis, and further validated the proliferation and migration functions of a candidate biomarker (i.e., AKR1B10).

## 2. Methods

### 2.1. Psoriasis Data and Principal Component Analysis

We downloaded the RNA-seq expression table from the National Center for Biotechnology Information Gene Expression Omnibus (GEO) data repository (http://www.ncbi.nlm.nih.gov/geo/). Dataset GSE54456 included RNA-seq samples from 92 lesional tissues from psoriasis patients and 82 skin samples from healthy individuals [1]. Dataset GSE66511 contained the RNA-seq results from 12 lesional psoriasis patients, 12 nonlesional psoriasis patients, and 12 normal skin biopsies [10]. The raw read counts were normalized to RPKM values (reads per kilobase per million reads). Then, all gene expression values were log2 transformed, and genes with null values were removed from all samples. The principal component analysis was performed using the “prcomp” function from the “base” package in R project.

### 2.2. DEGs Analysis

The fold change of the gene expression level and corresponding *t*-test *p* values were calculated between the psoriatic tissues and normal skin. DEGs were defined as genes that fulfilled the criteria of a fold change value >1.5 and a *t*-test *p* value < 0.05. The Venn diagram showed overlapping DEGs from two datasets and was drawn using the “VennDiagram” package of R.

### 2.3. Pathway Analysis

For pathway analyses, 696 DEGs were used to query the KEGG database to determine the biological significance. The Fisher exact test was used in the selection of enriched pathways using a *p* value < 0.05, and at least 3 differentially expressed genes were involved in the pathway, as the criteria. The pathway enrichment analysis was performed using the “KEGG.db” and “KEGGprofile” packages in R project.

### 2.4. Functional Annotation

Gene-annotation analyses were performed with the DAVID web tool [21, 22]. The 696 DEGs were used to query the GO database to determine the biological functions of these DEGs. An enriched GO term was determined through a significant Fisher exact test (*p* value < 0.05).

### 2.5. Coexpression Analysis

A coexpression network was constructed for the differentially expressed gene related to lipid metabolism. Pearson's correlation coefficient was calculated between the differentially expressed gene of lipid metabolism and all quantified genes in each dataset. We employed a coefficient >0.9 or <−0.9 and adjusted the *p* value to <0.01 as the criteria to identify coexpressed gene pairs. Then, the coexpressed gene pairs that were identified in both dataset were selected.

### 2.6. Knockdown Assay and Quantitative PCR Validation

HACAT cells were seeded in a cell culture plate with 50–90% cell density; then, siRNA-HilyMax complex (GenePharma, Shanghai, China) was added to the cell culture well, and the plate was incubated at 37°C in a CO_2_ incubator for 4 h; finally, the medium was changed, and incubation continued for 48 h. The assay was divided into five groups: NC (control), Vector, siRNA-1121, siRNA-1239, and siRNA-452.

Total RNAs from cells were extracted using a Cell Culture and Tissue Total RNA Extraction and Preparation Mini Kit, according to the manufacturer's instruction. The quantity and quality of RNA were confirmed with a NanoDrop 1000. The primers were designed using Primer Premier 5.0 software and synthesized from Generay Biotech Co., Ltd. Quantitative real-time PCR were performed using the KAPA SYBR Green SuperMix PCR kit with the iCycler apparatus system (Bio-Rad) (Table 1). Relative gene expression was calculated using the 2^−ΔΔCt^ method.

### 2.7. Scratch Wound-Healing Assay

HACAT cells were seeded in 24-well plates with a density of 1 × 10^5^ cells per well and incubated overnight. The cells were then scratched with a 10 *μ*L tip at the bottom of the wells, and the floating cells were gently washed twice with the medium. Then, the cells were transfected with HilyMax (Vector), siRNA-1121, siRNA-1239, and siRNA-452 (Table 2). Each well was photographed with microscope, and the gap distance was calculated at 0 h and 16 h using Photoshop (Adobe Systems Incorporated, USA).

### 2.8. Proliferation Assay

Cell proliferation was assessed with the Cell Counting Kit-8 (CCK-8) (Dojindo Laboratories, Kumamoto, Japan). Briefly, HACAT cells were seeded on a 96-well microplate at a density of 3 × 10^4^ cells per well, and then the cells were transfected with HilyMax (Vector), siRNA-1121, siRNA-1239, and siRNA-452. The cells were cultured for 0 h, 24 h, and 48 h. Then, 5 *μ*L of CCK-8 solution was added to each well and incubated at 37°C for an additional 2 h. Optical density (OD) was determined at a wave-length of 450 nm.

### 2.9. Statistical Analysis

We used Student's *t*-test to filter the differentially expressed genes between the control and patient groups (fold change value > 1.5 and *t*-test *p* value < 0.05), and the Fisher exact test was used to determine significant and the enriched pathway. Statistical analyses were performed using GraphPad Prism 5 software (GraphPad Software, USA).

## 3. Results

### 3.1. Psoriasis Gene Expression Dataset and Principal Component Analysis

**Figure 1:**
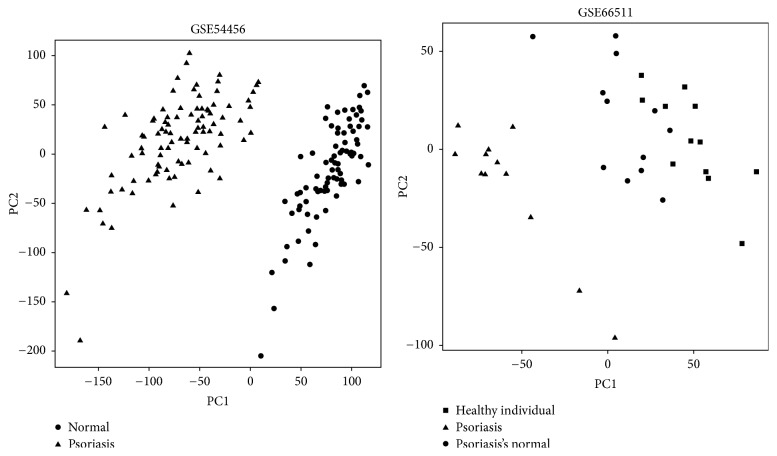
Principal component analysis shows that the psoriasis samples were clustered together and separated from normal samples. The nonlesional samples were clustered together with the samples of healthy individual.

To explore the molecular mechanism related to the pathogenesis of psoriasis, we obtained two datasets from the GEO database. Comparing the gene expression of psoriasis to that of normal skin allowed us to characterize the molecular mechanism underlying psoriasis initiation. The principal component analysis of the gene expression data provided an overall view of the variation between different sample types. As shown in Figure 1, the psoriasis and normal skins samples were clearly separated into two groups, according to the principal component analysis of gene expression. It may indicate the dramatic difference between psoriasis lesions and normal tissue in terms of their gene expression levels. In dataset GSE66511 [10], nonlesional samples of psoriasis and normal skin were sequenced, and these samples were clustered into one group, suggesting that the gene expression of nonlesional psoriasis is similar to normal skin of healthy individuals.

### 3.2. Differential Expression of Genes in Lesional Skin Compared to Normal Skin

**Figure 2:**
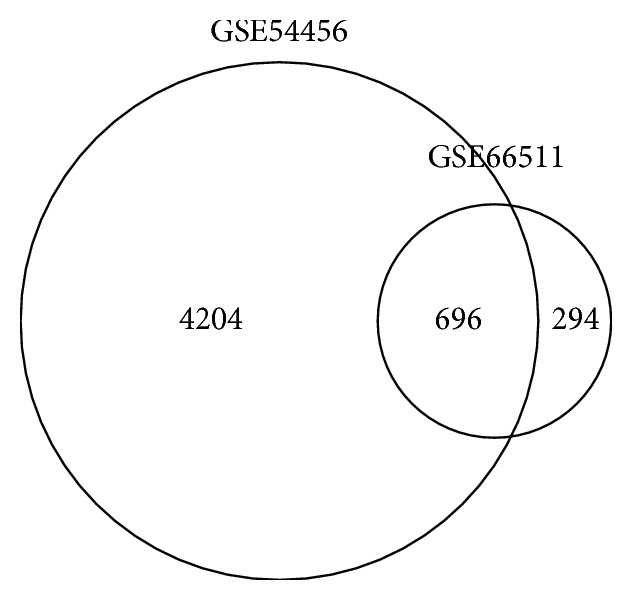
This Venn diagram shows the 696 genes identified as DEGs in both datasets.

Differentially expressed gene analysis was performed to reveal gene expression variations related to the pathogenesis of psoriasis. Gene expression fold change and *t*-tests were calculated by comparing psoriasis lesional tissue with the normal skin tissue in two datasets. DEGs were selected using the *t*-test criteria of a *p* value less than 0.05 combined with a fold change larger than 1.5. In total, 4,900 genes met the criteria in dataset GSE54456 [1], and 990 DEGs were identified in dataset GSE66511 [10]. We assumed that genes dysregulated in both psoriasis datasets play essential roles in the initiation and progression of psoriasis. The Venn diagram in Figure 2 shows that 696 genes were identified as DEGs in both datasets.

### 3.3. Functional Analysis of Differentially Expressed Genes

To reveal the biological process commonly changed in psoriasis, pathway enrichment analysis and GO annotation were performed. The 696 DEGs identified in both datasets were used to query the KEGG database and GO annotation database. The significantly enriched KEGG pathways with the Fisher exact *p* value < 0.05 are listed in Table 3. The top enriched pathway is the PPAR signaling pathway, in which 12 DEGs are involved. PPAR signaling pathway reportedly regulates lipid metabolism, including fatty acid oxidation, lipid homeostasis, and lipoprotein metabolism [23, 24]. Approximately 25 genes in the cytokine-cytokine receptor interaction pathway were significantly dysregulated in psoriasis. Most of these 25 genes belonged to the chemokine and interleukin gene families. We also observed that several linolenic acid and lipid metabolism pathways were significantly enriched, such as alpha-linolenic acid metabolism, linoleic acid metabolism, ether lipid metabolism, and glycosphingolipid biosynthesis pathways. The pathway enrichment analysis revealed that lipid metabolism pathways may play important roles in the pathogenesis of psoriasis.

To further characterize the biological function of 696 DEGs, the GO annotation results are listed in Table 4. The top three enriched GO terms were keratinization, ectoderm development, and keratinocyte differentiation. Previous studies have demonstrated that keratinization disorders were associated with retinoid metabolism in psoriasis [23–25], and epidermal development and keratinocyte differentiation were regulated by psoriasis [26]. Furthermore, 44 genes were involved in the immune response, and 17 genes related to the positive regulation of the immune system process were dysregulated.

### 3.4. Expression of Genes Related to Lipid Metabolism

The pathway analysis demonstrated the involvement of fat acid and lipid metabolism pathways in psoriasis. To further characterize the molecular mechanism, we took a detailed look at the gene expression alteration of these metabolism pathways in psoriasis. Nine genes were upregulated in psoriasis in both datasets. Notably, as shown in Table 5, the fold changes of AKR1B15 and AKR1B10 were extremely high in psoriasis dataset GSE54456. In addition, seven genes of the phospholipase A2 (PLA2G) gene families were significantly upregulated in psoriasis.

**Figure 3:**
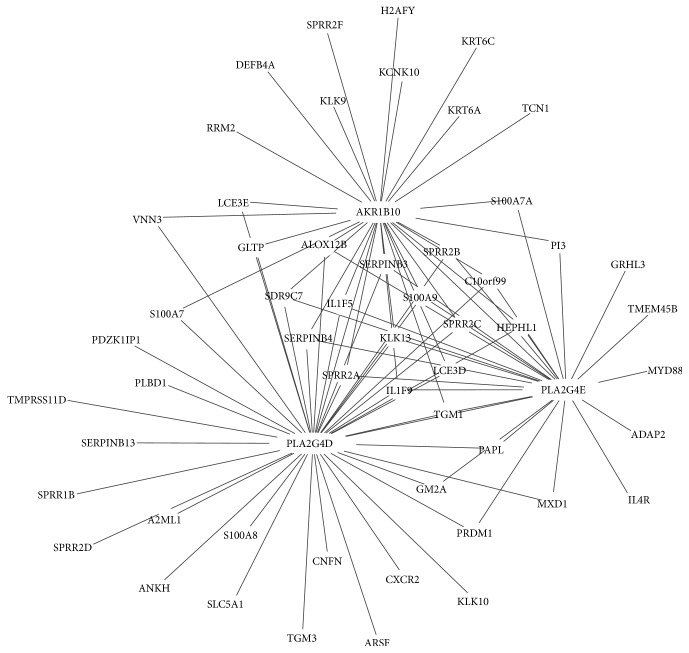
Coexpression network of lipid metabolism genes. Each point represents a gene, and lines indicate the interaction between two genes.

To investigate the important role of differentially expressed lipid metabolism genes, we constructed a gene-gene coexpression network. As shown in Figure 3, the network was composed of 3 hub genes (AKR1B10, PLA2G4D, and PLA2G4E). This coexpression network includes 95 gene-gene pairs. Among them, 13 genes were interacted with the three hub genes.

### 3.5. AKR1B10 Gene Expression Is Related to the Overproliferation and Migration of Keratinocyte

**Figure 4:**
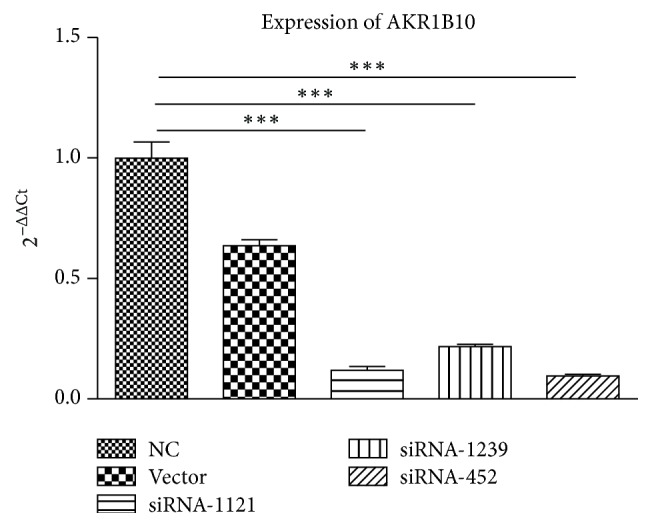
AKR1B10 expression after being transfected with HilyMax (Vector), siRNA-1121, siRNA-1239, and siRNA-452 in HACAT cells. The knockdown groups and the NC group (control) had statistically significance differences (*p* < 0.001). *∗∗∗* refers to *p* < 0.001.

We used an RNA interference experiment to further investigate the role of the AKR1B10 gene in the development and progress of psoriasis. The HACAT cell is a keratinocyte cell line from adult human skin and is commonly used to characterize the action of human keratinocytes. We knocked down AKR1B10 expression through siRNA candidates. As shown in Figure 4, the expression of AKR1B10 in the knockdown groups was significantly downregulated compared to the control groups (*p* < 0.001).

**Figure 5:**
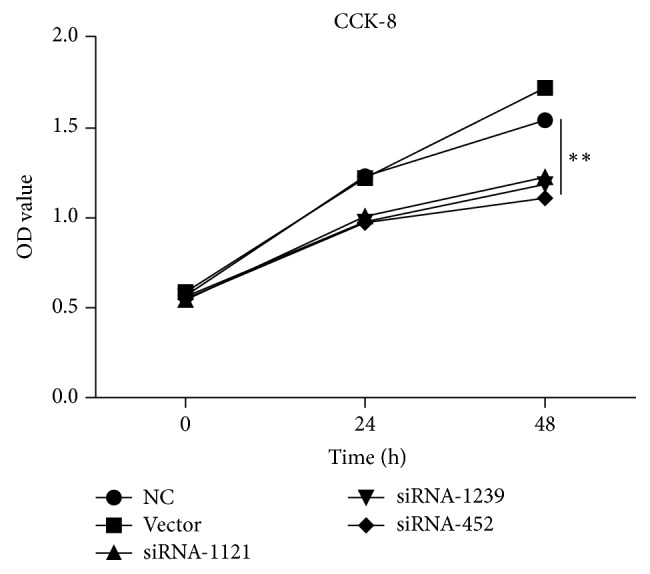
The proliferation of HACAT cells was evaluated with the CCK-8 assay. The results showed significant downregulation of cell proliferation between the knockdown groups and NC group (*p* < 0.01), and there is no significant difference between the NC and Vector groups. *∗∗* refers to *p* < 0.01.

The CCK-8 assays were used to estimate the effect of AKR1B10 gene knockdown on cell proliferation ability. As shown in Figure 5, significant downregulation of cell proliferation was observed between the knockdown and NC groups (*p* < 0.01), which suggested that the HACAT cells were inhibited by siRNAs. However, there was no significant difference between the NC and Vector groups.

**Figure 6:**
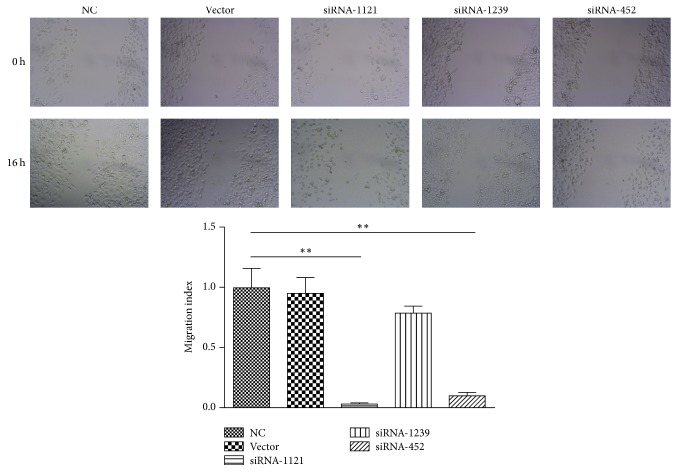
Scratch wound-healing assay. The migration ability of HACAT cells is inhibited by siRNA-452 (*p* < 0.01) and siRNA-1121 (*p* < 0.01) interference compared to the NC group. *∗∗* refers to *p* < 0.01.

Wound-healing assays were used to characterize the effect of AKR1B10 gene knockdown on cell migration ability. As shown in Figure 6, the results showed that, in contrast to the control group, a larger wound area remained uncovered following culture with siRNA-1121 and siRNA-452, which suggested that siRNA-1121 and siRNA-452 effectively blocked the migration of HACAT cells.

## 4. Discussion

Although current studies have identified many factors associated with psoriasis, such as immune disorders, sleep disorders [2], and disturbed hormone releases [3], several susceptibility genetic loci also contributed to the initiation of psoriasis [1]. However, the pathogeneses of psoriasis and lipid metabolism have not been completely revealed. In this study, we integrated two transcriptome expression profiles of psoriasis and presented a comprehensive differentially expressed gene analysis and function annotation. The analyses identified a list of genes that are overlapped and differentially expressed in both datasets, indicating the importance of these genes in psoriasis. Based on the pathway and GO annotation, we observed that the common DEGs of two datasets were involved in several lipid metabolism pathways and GO terms associated with the immune function. Notably, AKR1B10, AKR1B15, and several members of the PLA2 gene family were significantly overexpressed in psoriasis lesion tissue.

The PPAR signaling pathway was the top enriched pathway, and 12 genes involved in this pathway were significantly dysregulated in psoriasis. Previous studies have demonstrated that activation of PPARs could induce a psoriasis-like skin disease in vivo [24]. PPAR gene expression was significantly elevated in psoriatic patients, but it significantly decreased after treatment [27]. These studies demonstrated the important role of PPARs in psoriasis; therefore, PPARs could be promising targets in the treatment of psoriasis [28]. Interestingly, a report has shown that treatment with PPARs activators increased cholesterol, fatty acid, and lipid synthesis [29]. The PPAR agonists could activate epidermal lipid synthesis and transfer precursor lipids to lipids, which form the extracellular lipid membranes [30]. Furthermore, the lipid metabolism disorder associated with PPARs also contributed to the immunometabolism of psoriasis [8, 30].

The enrichment of alpha-linoleic acid metabolism, glycosphingolipid biosynthesis, and linoleic acid metabolism pathways further demonstrated the important role of lipid metabolism in psoriasis. In the two psoriasis datasets, PLA2 family members were significantly overexpressed. Our study suggested that PLA2 genes were significantly involved in the biologic function of psoriasis. Notably, the phospholipase A2 gene family members were significantly overexpressed in psoriasis. PLA2 is a superfamily of enzymes that catalyzes the production of free fatty acids and lysophospholipids [31]. A previous study has demonstrated that PLA2 was highly expressed in psoriatic lesions and also generated neolipid skin antigens for CD1a-reactive T cell recognition. Research also evidenced that overexpression of PLA2 contributed to lipid-specific CD1a-reactive T cells, which were associated with psoriatic inflammation [32].

Currently, AKR1B10 is rarely reported to be related to psoriasis and is only shown to play an important role in liver carcinogenesis and eating behavior [33]. We observed that this gene was the highest DEG expressed in the psoriasis tissue compared to normal tissue. Further functional analysis revealed that this gene was one part of the linoleic acid metabolism and implied that malfunction of the lipid pathway is associated with pathogenesis of psoriasis. We further validated its proliferative and migrating function through knockdown assays. Ma et al. have found that AKR1B10 is associated with tumor metastasis and also validated that AKR1B10 silencing resulted in the inhibition of cell growth, as well as the decrease of lipid synthesis, particularly phospholipids [34]. This result was similar to our findings, in terms of the molecular function of AKR1B10 (despite the different cell types). However, more functions of this gene are yet to be revealed, particularly how it works as a proliferation promoter.

In conclusion, our study partly demonstrated the association between lipid metabolism and psoriasis in terms of gene expression profiling. Not only was the highly expressed AKR1B10 gene involved in the malfunctioning linoleic acid pathway through data mining but it also induced overproliferation of keratinocytes through knockdown validation assays. This study provides a target for further therapeutic options for psoriasis.

## Figures and Tables

**Table 1:** Quantitative PCR primers sequence.

qPCR primer	Forward (5′ → 3′)	Reverse (5′ → 3′)	Product length
AKR1B10	CCCAGGTTCTGATCCGTTT	GCAACACGTTACAGGCCC	171 bp
GAPDH	GAGTCCACTGGCGTCTTCAC	TGCTGATGATCTTGAGGCTGTT	141 bp

**Table 2:** The synthetic siRNA sequence.

siRNA	Forward sequence (5′ → 3′)	Reverse sequence (5′ → 3′)
siRNA-1121	CACGCAUUGUUGAGAACAUTT	AUGUUCUCAACAAUGCGUGTT
siRNA-1239	GGAAGACUAUCCCUUCAAUTT	AUUGAAGGGAUAGUCUUCCTT
siRNA-452	GUGCCUAUGUCUAUCAGAATT	UUCUGAUAGACAUAGGCACTT

**Table 3:** Pathway enrichment analysis of 696 common DEGs.

Term	Count	%	*p* value	Genes
hsa03320: PPAR signaling pathway	12	1.7	1.10*E* − 04	ACOX2, LPL, PPARD, ACSL1, HMGCS2, PPARG, FADS2, FABP7, ACADL, SLC27A2, MMP1, FABP5
hsa04060: cytokine-cytokine receptor interaction	25	3.6	1.94*E* − 04	CXCL1, TNFRSF21, IL19, IL21R, CXCL9, CXCR2, CXCR3, KIT, IL7R, CCL20, CXCR4, IL4R, IL1B, IL6, IL18RAP, IL2RA, IL8, LIFR, EDAR, CCL18, IL20, CCR7, CCR5, CXCL13, EDA
hsa00592: alpha-linolenic acid metabolism	6	0.8	6.60*E* − 04	JMJD7-PLA2G4B, PLA2G2A, FADS2, PLA2G3, PLA2G4B, PLA2G4E, PLA2G2F
hsa00601: glycosphingolipid biosynthesis	6	0.8	3.22*E* − 03	FUT9, B3GALT5, FUT3, B3GNT3, FUT1, FUT2
hsa00591: linoleic acid metabolism	6	0.8	5.39*E* − 03	JMJD7-PLA2G4B, AKR1B15, AKR1B10, PLA2G2A, PLA2G3, PLA2G4B, PLA2G4E, PLA2G2F
hsa04640: hematopoietic cell lineage	10	1.4	9.08*E* − 03	IL6, IL2RA, CD3G, CD3D, CD3E, IL4R, CD2, IL1B, KIT, IL7R
hsa04062: chemokine signaling pathway	16	2.3	1.08*E* − 02	CXCL1, ITK, LYN, IL8, CXCL9, CXCR2, CXCR3, STAT1, CCL18, CCR7, PLCB4, CCR5, CCL20, CXCR4, CXCL13, JAK3
hsa00565: ether lipid metabolism	6	0.8	1.41*E* − 02	JMJD7-PLA2G4B, PLA2G2A, PLA2G3, PLA2G4B, CHPT1, PLA2G4E, PLA2G2F
hsa05340: primary immunodeficiency	6	0.8	1.41*E* − 02	CD3D, CD3E, ICOS, LCK, JAK3, IL7R
hsa00603: glycosphingolipid biosynthesis	4	0.5	1.85*E* − 02	FUT9, B3GALT5, FUT1, FUT2
hsa00564: glycerophospholipid metabolism	8	1.1	2.22*E* − 02	GPD1, JMJD7-PLA2G4B, PLA2G2A, PLA2G3, PNPLA3, PLA2G4B, CHPT1, PLA2G4E, PLA2G2F
hsa04730: long-term depression	8	1.1	2.39*E* − 02	JMJD7-PLA2G4B, PLCB4, GRIA2, LYN, PLA2G2A, PLA2G3, PLA2G4B, PLA2G4E, PLA2G2F
hsa00590: arachidonic acid metabolism	7	1.0	2.80*E* − 02	JMJD7-PLA2G4B, ALOX15B, PLA2G2A, ALOX12B, PLA2G3, PLA2G4B, PLA2G4E, PLA2G2F
hsa04514: cell adhesion molecules (CAMs)	11	1.6	4.78*E* − 02	CLDN16, CLDN8, CD80, NRXN3, SELL, ICOS, CD274, CD2, CTLA4, CDH2, CD226

**Table 4:** GO annotation results of 696 common DEGs.

Term	Count	%	*p* value	Genes
GO:0031424~keratinization	15	4.7	3.15*E* − 14	HRNR, LCE3A, LCE3D, SPRR2G, SPRR2F, LCE1B, SPRR2E, SPRR2C, SPRR2D, SPRR1B, SPRR2A, SPRR2B, CNFN, TGM1, TGM3, LCE3E
GO:0007398~ectoderm development	25	7.9	6.38*E* − 13	HRNR, KRT6A, KRT6B, LCE3A, S100A7, CRABP2, LCE3D, SPRR2G, LCE1B, SPRR2F, SPRR2E, SPRR2C, SPRR2D, SPRR2A, SPRR2B, TGM1, ALOX12B, TGM3, GRHL3, EDAR, KRT16, SPRR1B, CNFN, LCE3E, EDA, FABP5
GO:0030216~keratinocyte differentiation	16	5.0	1.35*E* − 12	HRNR, LCE3A, S100A7, LCE3D, SPRR2G, SPRR2F, LCE1B, SPRR2E, SPRR2C, SPRR2D, SPRR1B, SPRR2A, SPRR2B, CNFN, TGM1, TGM3, LCE3E
GO:0009913~epidermal cell differentiation	16	5.0	5.24*E* − 12	HRNR, LCE3A, S100A7, LCE3D, SPRR2G, SPRR2F, LCE1B, SPRR2E, SPRR2C, SPRR2D, SPRR1B, SPRR2A, SPRR2B, CNFN, TGM1, TGM3, LCE3E
GO:0006955~immune response	44	14.0	6.22*E* − 12	CXCL1, KYNU, S100A7, IL19, OAS3, RSAD2, OAS1, OAS2, DEFB4A, IL7R, PNP, NOD2, CCL20, RAET1G, HRH2, IL4R, BCL3, VNN1, IL1B, LTF, CCBP2, NOS2, FCGR3A, GBP6, IL18RAP, GBP5, LYN, IL8, CTLA4, CD300E, SLAMF7, AIM2, OASL, CCR7, APOL1, CST7, CXCL13, CD274, SERPINB4, CLEC7A, EDA, IFI6, PTAFR, GBP1
GO:0008544~epidermis development	23	7.3	7.26*E* − 12	HRNR, LCE3A, S100A7, LCE3D, CRABP2, SPRR2G, GRHL3, SPRR2F, LCE1B, SPRR2E, EDAR, SPRR2C, SPRR2D, KRT16, SPRR1B, SPRR2A, TGM1, SPRR2B, CNFN, ALOX12B, TGM3, LCE3E, EDA, FABP5
GO:0030855~epithelial cell differentiation	18	5.7	1.06*E* − 09	HRNR, LCE3A, S100A7, LCE3D, SPRR2G, SPRR2F, LCE1B, SPRR2E, RHCG, SPRR2C, SPRR2D, SPRR1B, SPRR2A, UPK1B, SPRR2B, CNFN, TGM1, TGM3, LCE3E
GO:0060429~epithelium development	19	6.0	3.98*E* − 07	HRNR, LCE3A, S100A7, LCE3D, SPRR2G, SPRR2F, LCE1B, SPRR2E, RHCG, SPRR2C, SPRR2D, FREM2, SPRR1B, SPRR2A, UPK1B, SPRR2B, CNFN, TGM1, TGM3, LCE3E
GO:0006952~defense response	31	9.8	2.58*E* − 06	CXCL1, KYNU, S100A8, S100A7, S100A9, RSAD2, CXCR2, DEFB4A, NOD2, CCL20, VNN1, IL1B, LTF, BCL3, SERPINA1, NOS2, MX1, IRAK2, IL18RAP, LYN, IL8, LYZ, SLAMF7, IDO1, S100A12, CCR7, APOL1, CXCL13, IRF7, CLEC7A, PTAFR
GO:0002684~positive regulation of immune system process	17	5.4	1.47*E* − 05	IRAK2, LYN, CD247, PTPN22, IDO1, IL7R, PNP, PRKCQ, NOD2, CD80, IL4R, CD2, IL1B, VNN1, CLEC7A, NOS2, CD226

**Table 5:** Genes related to lipid metabolism were dysregulated in both datasets.

	GSE54456		GSE66511	
	Fold change	FDR *p* value	Fold change	FDR *p* value
PLA2G4D	20.52	2.93*E* − 92	6.66	1.64*E* − 04
PLA2G4E	4.69	4.87*E* − 69	3.20	1.85*E* − 06
PLA2G2F	4.25	5.65*E* − 47	2.90	1.71*E* − 03
PLA2G3	4.19	2.10*E* − 60	2.86	8.65*E* − 04
PLA2G4B	3.70	1.26*E* − 57	2.72	1.30*E* − 07
JMJD7-PLA2G4B	2.43	1.54*E* − 44	2.65	4.29*E* − 08
PLA2G2A	2.12	1.28*E* − 06	6.11	1.29*E* − 03
*AKR1B10*	*78.39*	1.37*E* − 94	*12.52*	7.42*E* − 07
AKR1B15	60.88	2.03*E* − 45	2.80	1.31*E* − 03
